# Biomedical Informatics Techniques for Processing and Analyzing Web Blogs of Military Service Members

**DOI:** 10.2196/jmir.1538

**Published:** 2010-10-05

**Authors:** Sergiy Konovalov, Matthew Scotch, Lori Post, Cynthia Brandt

**Affiliations:** ^2^Department of Emergency MedicineYale UniversityNew Haven, CTUSA; ^1^Yale Center for Medical InformaticsYale UniversityNew Haven, CTUSA

**Keywords:** Blogging, medical informatics, military personnel, information storage and retrieval, combat disorders

## Abstract

**Introduction:**

Web logs (“blogs”) have become a popular mechanism for people to express their daily thoughts, feelings, and emotions. Many of these expressions contain health care-related themes, both physical and mental, similar to information discussed during a clinical interview or medical consultation. Thus, some of the information contained in blogs might be important for health care research, especially in mental health where stress-related conditions may be difficult and expensive to diagnose and where early recognition is often key to successful treatment. In the field of biomedical informatics, techniques such as information retrieval (IR) and natural language processing (NLP) are often used to unlock information contained in free-text notes. These methods might assist the clinical research community to better understand feelings and emotions post deployment and the burden of symptoms of stress among US military service members.

**Methods:**

In total, 90 military blog posts describing deployment situations and 60 control posts of Operation Enduring Freedom/Operation Iraqi Freedom (OEF/OIF) were collected. After “stop” word exclusion and stemming, a “bag-of-words” representation and term weighting was performed, and the most relevant words were manually selected out of the high-weight words. A pilot ontology was created using Collaborative Protégé, a knowledge management application. The word lists and the ontology were then used within General Architecture for Text Engineering (GATE), an NLP framework, to create an automated pipeline for recognition and analysis of blogs related to combat exposure. An independent expert opinion was used to create a reference standard and evaluate the results of the GATE pipeline.

**Results:**

The 2 dimensions of combat exposure descriptors identified were: words dealing with physical exposure and the soldiers’ emotional reactions to it. GATE pipeline was able to retrieve blog texts describing combat exposure with precision 0.9, recall 0.75, and F-score 0.82.

**Discussion:**

Natural language processing and automated information retrieval might potentially provide valuable tools for retrieving and analyzing military blog posts and uncovering military service members’ emotions and experiences of combat exposure.

## Introduction

Web logs (“blogs”) have become a popular mechanism for people to express their daily thoughts, feelings, and emotions. Much of the information contained in blogs includes health-related themes, both physical and mental. The matters described in a blog post may be very similar to the information discussed during a clinical interview or psychological consultation. Therefore, blogs might contain information that could be important for clinical research, especially in the mental health field where stress-related symptoms are often difficult to ascertain or measure and where timely recognition is often key to successful therapy [1].

In the field of biomedical informatics, information retrieval (IR) techniques (such as automated indexing) and natural language processing (NLP) are commonly used to unlock information contained in free narrative-style text notes [2-4]. With a focus on blogs, these methods can support “infoveillance” [5] and assist the clinical research community to better understand feelings and emotions post deployment and the burden of symptoms of stress among US military service members. In this study, we sought to evaluate the potential for using available IR and NLP tools to unlock information in blogs related to US military service members’ experiences and emotions of combat exposure by analyzing the blogs of the military personnel deployed during Operation Enduring Freedom/Operation Iraqi Freedom (OEF/OIF).

## Methods

### Blog Selection

The authors evaluated military blogs available on the Internet and manually selected 90 blog posts describing combat exposure according to selection criteria. Military blogs catalog Milblogging.com [6] lists over 2500 English-language military blogs and provides basic categorization functionality (sorting by location, time, popularity etc). Three authors (SK, MS, CB) independently used the ordered list of blogs from Iraq and Afghanistan in the period of 2002 to 2008 from this site and selected 1 to 3 blog posts from each site that (1) were in the English language, (2) contained a first person description of the events, (3) were not less than 5 sentences long, (4) were not written for business purposes (eg, news piece or military report), and (5) described concrete situations and events related to active duty.

Of the collected blog posts describing combat exposure, 50 were used for indexing and term weighting and 40 for the pilot evaluation and analysis.

Two of the authors (CB, MS) selected 60 blog posts from Milblogging.com conforming to the same selection criteria listed above except that they did not necessarily describe combat exposure. These 60 blogs were used as a control set during evaluation.

### Indexing and Term Weighting

A “bag-of-words” representation was employed to analyze and categorize the blog texts. In this model, the word order is not taken into consideration, and each text is treated as simply a collection of words. Such text representation is used in most typical approaches to text classification [7].

Python version 2.6.2 [8] with Natural Language Toolkit (NLTK) version 2.0b6 [9] was used for data preprocessing and term weight calculation. Data preprocessing included tokenizing (breaking up the texts into separate words), stemming, and removal of “stop” words. Stemming is the process of reducing morphologically related words to their common base form. Alternate spellings were treated as unique words during the stemming phase. The stop word list was created in order to automatically discard common words, that is, stop words, that by definition do not have any combat-specific meaning (articles, prepositions, and words such as go, do, have).

Term weighting is a standard procedure used in automated indexing to assess the importance of individual words in a corpus of documents [10]. We used “TF*IDF” term weighting in order to evaluate word frequency distribution and assess the frequency of occurrence of the words related to combat exposure. In this approach, the indexing weight is a product of term frequency (TF) and inverse document frequency (IDF). TF is a measure of the frequency of a word across a set (corpus) of documents, and IDF is a measure of the frequency of a word within a given document.

### Ontology Creation and Use

The resulting frequency distribution tables were reviewed by three authors (CB, MS, SK) in order to select the words relevant to combat exposure used in blog posts. Upon discussion and consensus from all authors, the list of relevant words was created. After that, the selected words were used in creation of a pilot ontology. An ontology is a way to create formal definitions of concepts by specifying semantic relationships between these concepts [11]. Creation of the ontology is an important step for future research as several open-source NLP suites have ontology-aware tools. Collaborative Protégé (version 3.4.4, build 579) [12], an ontology editor, was used to create and store the ontology.

The ontology was imported into General Architecture for Text Engineering (GATE) NLP framework (version 5.2.1, build 3581) [13]. GATE is one of several available open-source NLP tools. The ontology and word lists were used to construct ontology-based gazetteers: alphabetized word lists that are used by GATE for text analysis. The ontology annotation tool, Java Annotation Patterns Engine (JAPE) [14], was then applied to further refine text tagging. JAPE grammar is a set of (modifiable) rules that determine actions that are implemented during the annotation [14].

### Pilot Evaluation

NLP performance was evaluated by an author (LP), who is an expert in the field of evaluation. Here, focus was on whether our system was able to identify blogs related to the deployment experience (and therefore of interest to the health care provider) from a larger set of texts. Selected posts from the combat exposure set and the control set were run through the GATE pipeline and at the same time reviewed by the fourth author (LP), who was previously unfamiliar with the ontology and word lists but who has had extensive experience in clinical work with military service members. The blog posts were categorized by this author into 2 groups: those that did and did not describe clinically relevant combat exposure. The results of the GATE pipeline on the same blog posts were then evaluated in reference to this clinical categorization (ie, the “expert standard”). Standard information retrieval metrics in biomedical informatics literature such as precision, recall, and F-score were used for the evaluation [15,16].

## Results

After stemming and stop words exclusion, the indexing weights of all word stems in blog posts were calculated. We selected 263 word stems related to combat exposure out of those which had indexing weight above 0.1. The 20 most frequent word stems (those with the largest weight) are shown in Table 1.

**Table 1:** Most frequent word stems related to combat exposure

Word Stem	Weight	Index Document Frequency (IDF)
Explos	1.38	0.71
Mission	1.37	0.72
Truck	1.37	0.72
Soldier	1.24	0.82
See	1.20	0.85
Fire	1.17	0.87
Patrol	1.15	0.89
Vehicl	1.08	0.94
Hit	1.05	0.96
Attack	1.05	0.96
Stop	1.05	0.96
Deton	1.00	1.0
Bomb	0.97	1.02
Secur	0.91	1.07
Mortar	0.91	1.07
Deploy	0.88	1.10
Shot	0.84	1.12
Happen	0.81	1.15
Weapon	0.81	1.15

### Combat Exposure Dimensions

Words related to combat exposure events had 2 different dimensions: (1) characteristics of the physical exposure itself such as firing a weapon, being attacked, or witnessing an explosion (eg, “explosion,” “bomb,” “shot”) and (2) feelings and emotions associated with the exposure such as being scared, feeling compassion (eg, “secure,” “hope”). The most frequent words were manually separated into the 2 categories. These dimensions were then incorporated into the ontology structure.

**Figure 1:**
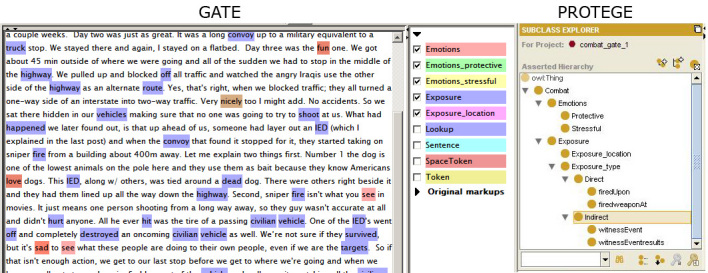
Use of ontology in GATE

Within the ontology we created separate categories for the physical combat exposure and emotions associated with it. Under the exposure category, 2 subcategories were created: direct and indirect. We defined direct exposure as an active involvement in a combat situation (such as firing a weapon), while an example of an indirect exposure would be witnessing a combat-related event. We also created 2 subcategories for the emotions: protective (described by words such as “hope,” “safe,” “supporting”) and stressful (such as “scared,” “upsetting,” “nervous”). Figure 1 shows the ontology we created and how the ontology was used to annotate a sample blog in GATE. Note that on the left in Figure 1 is a GATE screenshot with color-coded ontology categories. The combat-related works in the text are annotated in accordance with the ontology (purple for physical exposure, and pink and brown for emotions). On the right in Figure 1 is a Protégé screenshot with ontology.

### Pilot Evaluation

We ran 20 randomly selected blog posts through our GATE pipeline and calculated the number of annotations (ie, the number of times words from our ontology were highlighted). For each document, the ratio of the number of annotations to the total word count (N_ann_/N_wc_) of the document was used as a measure of “richness” of the text in terms of the words related to combat exposure. We took the median of the ratios for all posts (4.95%) as a threshold. A given post was said to be recognized as relevant to combat exposure when the ratio N_ann_/N_wc_ fell above the threshold and irrelevant if the ratio fell below the threshold.

**Table 2:** Classification of blog text by relevancy to combat exposure by expert opinion and by our GATE pipeline for which the threshold is 4.95%

	Relevance of Text to Combat Exposure (Italics = Relevant, Nonitalics = Irrelevant)	
Post	According to Expert Opinion	According to GATE Pipeline (N_ann_/N_wc_)
Post 1	*Yes*	4.59%
Post 2	No	*5.05%*
Post 3	No	4.31%
Post 4	No	4.18%
Post 5	No	2.80%
Post 6	No	4.46%
Post 7	*Yes*	*8.47%*
Post 8	No	3.03%
Post 9	*Yes*	*6.83%*
Post 10	*Yes*	4.84%
Post 11	*Yes*	*9.09%*
Post 12	*Yes*	*6.74%*
Post 13	*Yes*	*7.63%*
Post 14	No	4.73%
Post 15	*Yes*	*7.09%*
Post 16	*Yes*	*8.15%*
Post 17	*Yes*	3.81%
Post 18	No	3.44%
Post 19	*Yes*	*7.77%*
Post 20	*Yes*	*6.83%*

Compared to the expert opinion (“reference standard”) our system returned 9 true positives, 1 false positive, 7 true negatives, and 3 false negatives. Precision is defined as (TP/(TP+FP)) and is the fraction of documents retrieved that are actually relevant to the search criteria. Recall is defined as (TP/(TP+FN)) and is a measure of sensitivity, ie, the fraction of all possible relevant documents in the test set that are retrieved. F-score is a harmonized mean of precision and recall, and provides an overall measure of the effectiveness of an information retrieval system. Our GATE pipeline performed with a precision of 0.9, recall of 0.75, and F-score of 0.82.

### Examples

**Figure 2:**
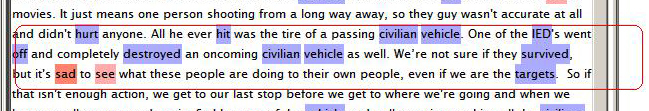
A section of an annotated blog describing the trauma of war

In the highlighted passage in Figure 2 many words related to combat exposure (purple) are found within 1 or 2 sentences of words related to emotions and senses (red, pink).

This paragraph presents an example of a specific traumatic factor: an attack against a soldier that injured innocent civilians instead.

**Figure 3:**
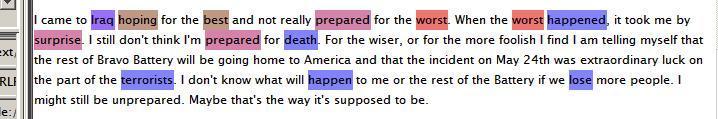
A section of an annotated Web blog showing physical and emotional descriptors

Another example of the proximity of the words related to the 2 dimensions of combat exposure can be seen in the annotated paragraph in Figure 3. The purple labels mark the combat events and locations, while the red and brown mark the words from the emotions category.

This passage follows the description of learning that some of the fellow soldiers were killed during convoy and clearly shows the deep emotional response to this event.

## Discussion

### Findings

We identified 2 dimensions of combat exposure descriptions: physical and emotional. These have been incorporated into the ontology and given specific annotations in GATE. The results of the pilot evaluation show that even without taking context into account our pipeline was able to retrieve the relevant blog posts with relatively good precision and recall.

We found that many of the words related to concrete experience and emotional reaction occurred within one or two sentences of each other frequently pointed to the paragraphs in the text that described the most dramatic combat situations. This may be instrumental in text analysis and uncovering the burden of combat exposure. This finding will be important for future research that will include context-aware analysis.

### Challenges

Blog texts are a heterogeneous and, at times, poorly structured text material. This distinguishes them from more structured texts like radiology reports and makes text processing more challenging. Apart from typos and spelling errors, there are field-specific abbreviations, slang terms, and intentional spelling variants. For example, one of the commonly seen words was an exclamation “Boom!” that described an experience of sudden loud sound, such as explosion; this word came in many different shapes, such as “BOOM,” “Boooom,” and “Bo-o-om.” One of the commonly encountered words was an abbreviation IED, which stands for improvised explosive device; this word was also often misspelled (“IOD,” “IUD” etc). The analysis of context (such as negation and word proximity) in blogs will also be more difficult because of poor structure and the informal nature of the blog text.

### Limitations

Although we found the bag-of-words weighting approach to be useful in discovering the vocabulary used by blog writers to describe combat situations and experiences, the adequacy of this approach for blogs remains a point of discussion. The bag-of-words approach is restrictive as it does not allow analyzing words in context. Thus, semantic ambiguity is difficulty to address with this approach. Other researchers have found similar limitations to this type of approach due to issues such as scarcity of data [17]. However, a potentially more informative context-aware mechanism (“rich bag-of-words”) is not possible to construct without a domain-specific vocabulary that we will be able to create using the results of the basic bag-of-words algorithm. This work is the first step toward creating a vocabulary for future context-aware tools.

There are other important limitations to this work. First, the generalizability of the sample may be questioned because of an important selection bias: by analyzing the blogs one can only know the experiences and opinions of people who have blogs. This is especially concerning in military blog analysis, because not every soldier at all times has access to the Internet even if they desire to have a blog and are allowed to do so by their commanding officers. Nevertheless, blogs can be used as a general measure of combat stress and may also be used to help identifyand quantify novel stressors specific to modern wars (for example, IEDs and suicide bombs). Another issue is that blogs represent a form of retrospective self-report and are by nature less reliable than the more objective methods of external surveillance such as videotaping or clinical interview. The external methods, however, are rarely available at the battlefield or are too expensive to be considered. Moreover, the consequences of combat stress may differ in their severity depending upon the attitudes and predeployment neurocognitive functioning of the person who experienced it [18].

By analysis of the blogs it was not possible to reliably identify the authors of the blogs who were active duty soldiers. In fact, the nature of the “blogsphere” is such that one cannot be sure whether the author of the blog really exists or is a fictional character. In addition, accurate assessment of temporality is a challenge in blogs. It is not always clear when the soldier has described a particular experience, whether it was right after the events or after some time upon recollection. However, given that a blog post describes actual combat events from the first person, it is still possible to use this blog post in the analysis, because the language used is similar. An additional issue found was that a portion of the blogs retrieved by Internet search seemingly conforming to the search criteria and using military language proved to be descriptions of the video game experience. It is thus essential to design an NLP system that accurately identifies these posts as being negative for combat exposure.

Another limitation is that the type of content and the personal details that are discussed in the blog may differ substantially from those mentioned during a person-to-person clinical interview.

### Possible Alternatives

Our approach to blog analysis was based on lexicons and ontologies created manually, which is both time-consuming and domain-specific. An alternative to this could be unsupervised or semisupervised approaches that have been applied to online reviews and blogs analysis [19]. Other alternatives to the GATE pipeline are possible, such as Unstructured Information Management Architecture (UIMA) [20], which is also open source.

There are ways to analyze natural text without creation of an ontology. However, ontologies play an important role in knowledge management and data integration [21]. Having the data structured by way of ontology will help us in the future research that will involve optimization of the existing algorithm and development of context-aware analysis. Many available open-source NLP systems now include ontology-aware tools; creation and development of the ontology will make the knowledge obtained from our study transferable.

### Possible Applications and Future Research

In the course of our study, we have encountered a multitude of blog posts of the military genre that had nothing to do with combat exposure. Selecting the relevant posts manually was a lengthy and tedious task. Our GATE pipeline, especially when evolved into a more context-aware tool, could be used for automated selection of blog posts (or diary entries, or even physician’s notes) that discuss combat exposure from all available text data. This can be instrumental in clinical work as it may help therapists and researchers to concentrate on the text material relevant to the combat exposure.

Our present work is a first step toward creating a more comprehensive context-aware algorithm that can be used for large-scale blog text categorization and analysis. Without the context analysis (such as negation, word order, and proximity) and more sophisticated software framework, it will not be possible to analyze thousands of documents or correctly classify them by combat exposure descriptions.

When developed to the point where the analysis of many thousands of documents is possible, an application such as ours could be used as a training tool for psychiatrists who are working with military personnel. It is also possible that an NLP application like this one may be used in clinical work to provide clinicians with text material discussing combat exposure and deployment-specific traumatic factors.

Future research is needed to demonstrate possible correlations between a person's descriptions of his or her wartime experiences in their blog with the ensuing symptoms or disorders. Focus groups and medical records analysis could be used for this purpose. The information obtained from military blogs may also be used during the focus groups to facilitate discussion and elucidate difficult or unusual subjects.

We conclude that available open-source natural language processing and automated information extraction tools may be instrumental in the analysis of free text contained in blogs. Analysis of military blog posts available on the Internet may help uncover the military service members’ emotions and experiences of combat exposure.

## Abbreviations

GATE: General Architecture for Text Engineering
HSRD: Health Services Research and Development
IED: improvised explosive device
IR: information retrieval
IDF: inverse document frequency
JAPE: Java Annotation Patterns Engine
NLM: National Library Of Medicine
NLP: natural language processing
OEF/OIF: Operation Enduring Freedom/Operation Iraqi Freedom
TF: term frequency
UIMA: Unstructured Information Management Architecture
